# Supplementary figures and images for: Microbial Detoxification of Sediments Underpins Persistence of Zostera marina Meadows
Source: Int J Mol Sci. 2024 May 16;25(10):5442. doi: 10.3390/ijms25105442 (PMC11122150; doi:10.3390/ijms25105442)

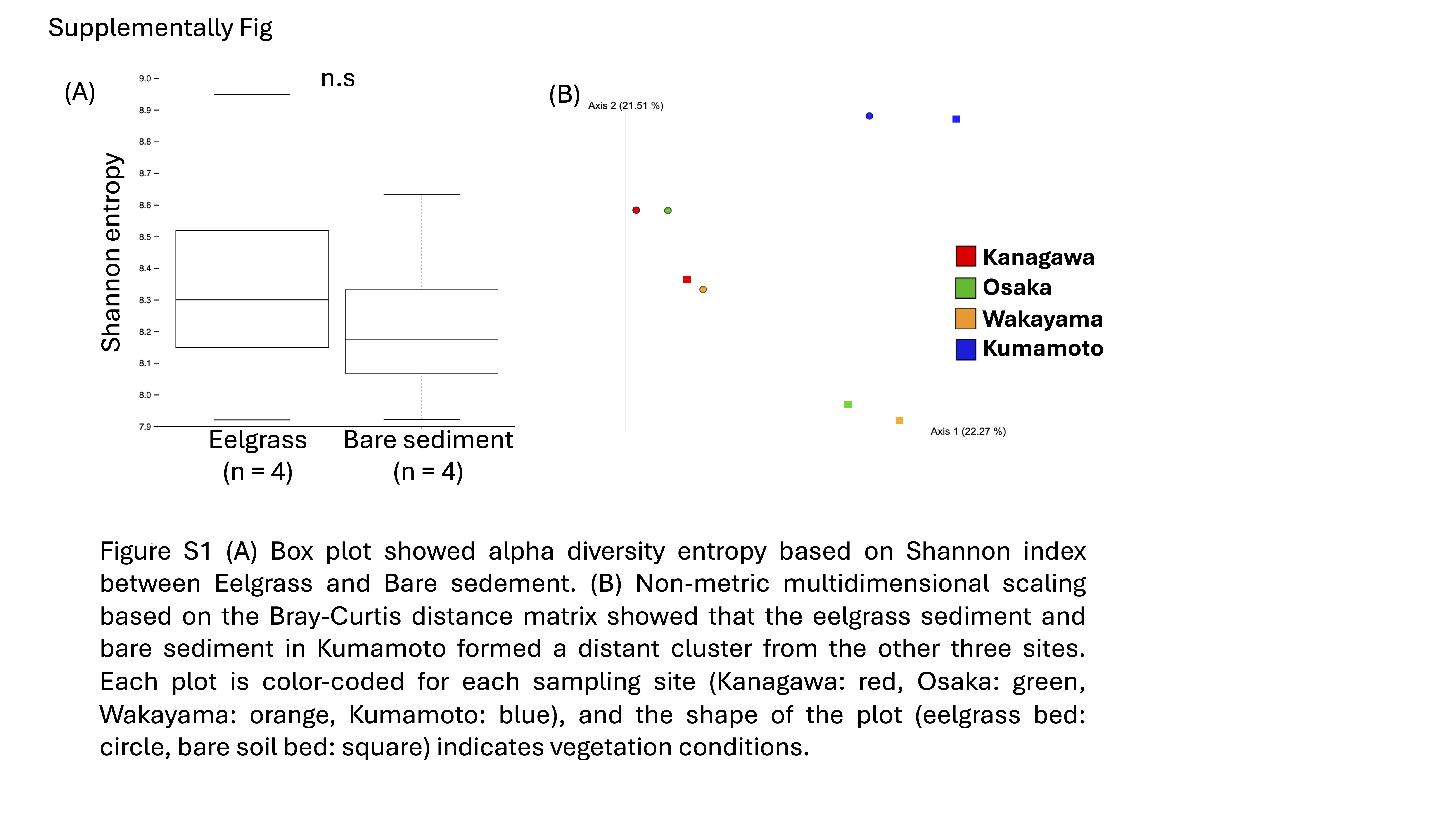

Supplement: Supplementary file 1 [file ijms-25-05442-s001.zip › Figure S1.jpg]

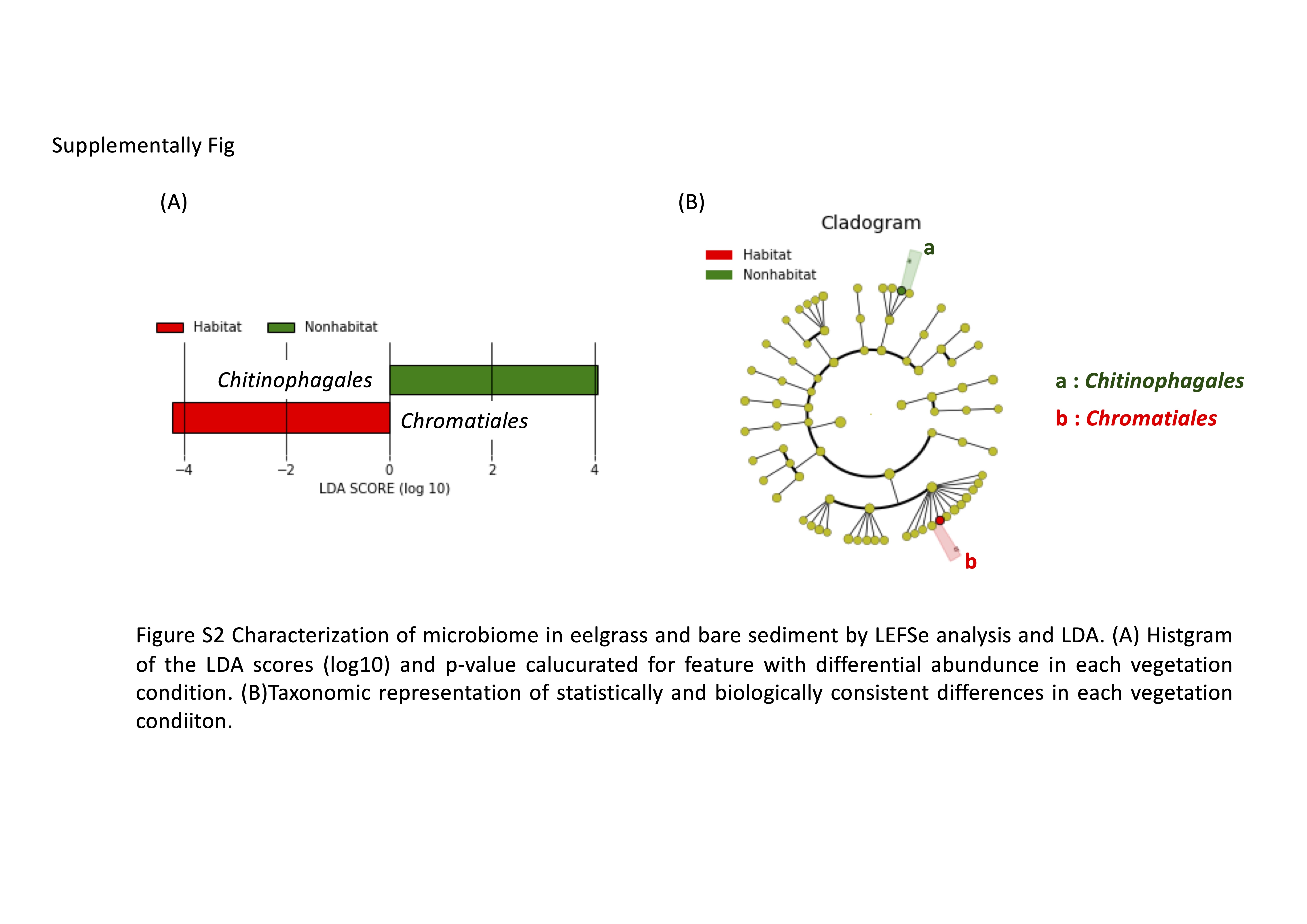

Supplement: Supplementary file 1 [file ijms-25-05442-s001.zip › Figure S2.jpg]

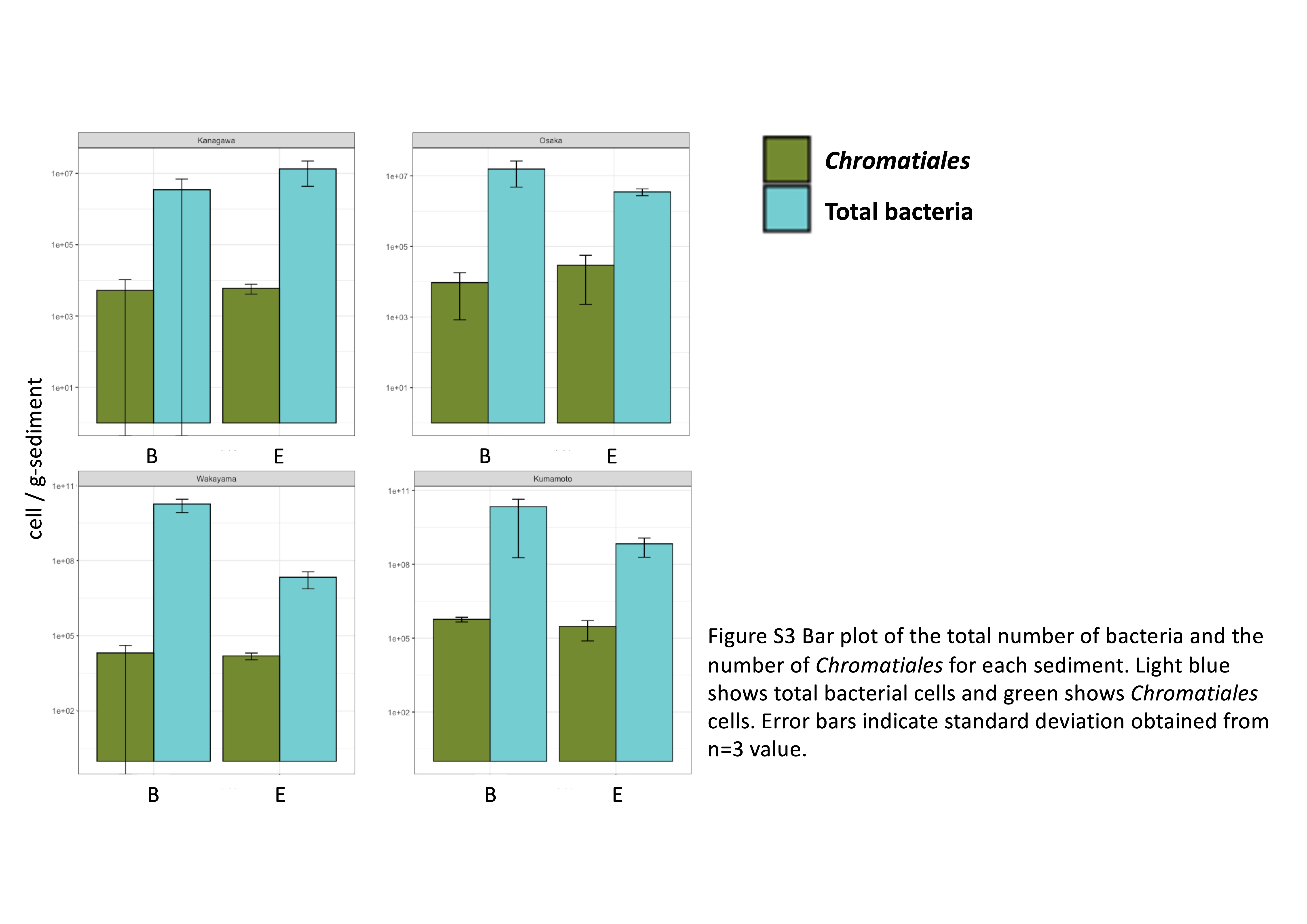

Supplement: Supplementary file 1 [file ijms-25-05442-s001.zip › Figure S3.jpg]
